# Lung Cancer Heterogeneity in Modulation of Th17/IL17A Responses

**DOI:** 10.3389/fonc.2019.01384

**Published:** 2019-12-10

**Authors:** Dominique Armstrong, Cheng-Yen Chang, Donald R. Lazarus, David Corry, Farrah Kheradmand

**Affiliations:** ^1^Department of Medicine, Baylor College of Medicine, Houston, TX, United States; ^2^Center for Translational Research on Inflammatory Diseases (CTRID), Michael E. DeBakey Department of Veterans Affairs, Houston, TX, United States; ^3^Department of Pathology and Immunology, Baylor College of Medicine, Houston, TX, United States; ^4^Biology of Inflammation Center, Baylor College of Medicine, Houston, TX, United States

**Keywords:** tumor heterogeneity, lung cancer, immune landscape, tumor microenvironment, immunotherapy, Th17, IL17A

## Abstract

The interplay between tumors and their immune microenvironment is critical for cancer development and progression. The discovery of tumor heterogeneity has provided a window into a complex interplay between tumors, their secreted products, and host immune responses at the cellular and molecular levels. Tumor heterogeneity can also act as a driving force in promoting treatment resistance and correlates with distinct tumor-mediated acquired immune responses. A prevailing question is how genetic aberrations in solid tumors can shape the immune landscape, resulting in pro-tumor or anti-tumor activities. Here we review evidence for clinical and pathophysiological mechanisms that underlie different types of non-small cell lung cancer (NSCLC) and provide new insights for future immunomodulatory-based therapies. Some of the more common driver mutations in NSCLC heterogeneity includes the opposing immune responses in oncogenic mutations in *K-ras* vs. non-*K-ras* models and their pro-inflammatory cytokines such as interleukin (IL)17A. We will discuss possible molecular and metabolic mechanisms that may govern the opposing immune responses observed in distinct genetic models of NSCLCs. A deeper understanding of how tumor heterogeneity modulates immune response can improve current therapeutic strategies and provide precise treatment to individual lung cancer patients.

## Introduction

Lung cancer is the leading cause of cancer-associated deaths, and its predominant histological presentation (e.g., adeno- and squamous-carcinoma) are collectively referred to as non-small cell lung cancers (NSCLCs) (1). Several mutations have been discovered in association with NSCLC development, and are used to characterize high interpatient heterogeneity (2). The tumor microenvironment is also highly heterogeneous because it includes different mutated cancer cells, and a diverse array of immune cells collected in and around solid tumors (3). Paradoxically, immune cells in the tumor microenvironment can promote tumor survival or destroy transformed cells, providing both pro- or anti-tumor responses, respectively (3). Mediators of these processes include cytokines, chemokines, metabolites, and checkpoint ligands. Thus, the immune cell milieu and the tumor microenvironment plays a vital role to eliminate tumor cells. Tumor cells that manage to escape the immune surveillance propagate and invade the surrounding tissue. Adaptive immune cell infiltration has been described in several type of solid tumors, including T helper 17 (Th17) cells (4), a subset of CD4^+^ cells which secrete IL17A. Th17 cells are recruited to tumor sites and inflammatory sites by C-C motif chemokine ligand 20 (CCL20) expressed by epithelial and stromal cells, interacting with C-C Chemokine receptor type 6 (CCR6) on Th17 cells (5, 6). The role of Th17 cells in promoting or inhibiting various human cancers seems to be context-dependent (7, 8). The influence of environmental cues may be one of the major determinants to modulate Th17 cell recruitment and function. Recently, growing evidence indicates that tumor-intrinsic genetics determine the corresponding immune profile. To further understand how tumor mutations impact Th17/IL17A response, we review the role of this response in human tumors as well as the studies in *K-ras* vs. non-*K-ras* driven NSCLC (9–11). We also discuss immunotherapies and offer possible molecular and metabolic mechanisms that modulate Th17 cells in tumors.

## Association Between Th17/IL17A in Human Tumors

### Pro-Tumor Effect of IL17A

Chronic inflammation is one of the hallmarks of malignant transformations (3). Induction of IL17A to various inflammatory conditions promotes the recruitment of innate immune cells such as neutrophils, and macrophages (12). Cigarette smoking which is associated with over 80% of all lung cancers, recruits Th17 cells in the lungs (13, 14), and has been associated with poor survival in NSCLC patients (15). Serum IL17A level is positively linked to vascular endothelial growth factor (VEGF) concentration in NSCLC patients, suggesting IL17A may promote angiogenesis in the tumor (16). Further, patients with high serum IL17A concentrations demonstrated a shorter overall survival rate compared with those with low levels (17). High IL17A levels also correlated with increased lymph node invasion, and distant metastases in NSCLC (17). Several meta-analyses have shown that high IL17A expression prognosticated poorer survival outcome or late stage diagnosis in NSCLC patients (18–20). Th17 cell infiltration also positively correlated to poor prognostic outcome in several other types of cancer, including colon, gastric, and liver. In contrast, Th17 cell infiltration in ovarian cancer has been shown to associate with better survival (21), while in nasopharyngeal cancer patients there was no significant association between tumor-infiltrating Th17 cells and survival, indicating a specific role for Th17 cells based on the specific tumor (22).

In addition to Th17 cells and association with tumor survival, chemokines and their receptors related to trafficking of this T cell subset have also been examined in NSCLC. For instance, high expression of CCR6, a chemokine receptor expressed by Th17 cells (23), was associated with shorter disease-free survival in NSCLC patients. Similarly, CCL20, the only chemokine known to interact with CCR6 (24), was elevated in the tumor compared to tumor-free adjacent lung tissue (25). Together, these findings suggest that the CCL20/CCR6 axis might facilitate infiltration of Th17 cell in NSCLC and promote tumor progression (25).

In addition to IL17A, Th17 cells can also secrete other cytokines, including IL-22 (26). Although elevated IL-22 expression has been detected in the primary lung tumor, serum, and malignant pleural effusion in patients (27, 28), its expression did not correlate with prognostic outcome in smokers with NSCLC (27). Further, IL-23, another cytokine that is secreted by myeloid cells and can polarize naive CD4^+^ T cells to Th17 cells (29, 30), was found to be elevated in the serum of lung cancer patients compared with healthy controls (31). Similarly, however, there is no known correlation between IL-23 expression and NSCLC prognosis to date.

### Anti-tumor Effect of IL17A

Multiple lines of evidence suggest that IL17A/Th17 may play a pro-tumorigenic role as an increased number of Th17 cells are found in human colorectal (32), gastric (33), hepatocellular (34), and lung cancers (35). However, despite the aforementioned-association studies, recent evidence also suggests the possibility for an immuno-protective role of Th17 cells in tumors. Different subsets of TILs in NSCLC can produce IL17A, such as natural killer, natural killer T cells, and γδT cells (36) but CD4^+^ stem cell-like memory T cells showed the highest expression of this cytokine (37). Since Th17 cells could transdifferentiate into interferon-gamma (IFN-γ)-secreting Th1 cells (38, 39), increased Th17 cells infiltrate into the tumor may promote tumor regression. In support of this concept, induction of Th17 cells to IFN-γ-secreting Th1 cells and differentiation into a durable stem memory phenotype enhances long-term anti-tumor responses (37, 40), and this feature has been applied in adoptive T cell transfer therapy in a murine preclinical model (41).

Targeting Th17 or IL17A pathways as a treatment for cancer has not yet been reported in clinical trials; however, two recent reports suggest their important role in anti-tumor activity. In one case, humanized monoclonal anti-IL17 treatment of psoriatic lesions in a patient with colon cancer was associated with cancer relapse after initial successful therapy with anti-programmed cell death protein 1 (PD-1) (42), though it is not clear whether this patient would have relapsed without the depletion of IL17A. Another case showed that anti-PD-1 treatment in patients with melanoma increased Th17 cell numbers in responders compared to non-responders (43). Although the role of Th17 cells in checkpoint blockade treatments remains unknown, these clinical reports highlight a potential anti-tumor effect of Th17 cells in immune-targeted therapy.

## Genetic Determinants of Th17 Response

Th17 cells have been shown to have both pro- and anti-tumor effects. Their action depends on the intrinsic and extrinsic phenotypes of the tumor milieu. Given that Th17 cells express other factors, one explanation for the opposing roles of Th17 cells in different cancer genomics may be their cellular heterogeneity. The different tumor genetics may also induce different subsets or phenotypes of Th17 cells that leads to their pro- or anti-tumor roles. Studies have shown that IL-23 induces TGFβ3 rather than TGFβ1, which combined with IL-6 leads to development of pathogenic Th17 cells, which are both functionally and transcriptionally distinct from homeostatic, non-pathogenic Th17 cells induced by TGFβ1, as shown in experimental autoimmune encephalitis models (44, 45). Additionally, there are other microenvironmental factors, such as specific microbial species in the intestine, that have been shown to induce pathogenic Th17 cells (46), whereas commensal bacteria can maintain homeostatic, protective Th17 cells in the gut (46, 47). Thus, there may be other inducers of pathogenic or homeostatic Th17 cells at work in these lung tumor models, perhaps through secreted cytokines by the tumor cells. However, more research is necessary to elucidate which types of Th17 cells are present in each of these models and to understand different subtypes of Th17 in different conditions as well as their role in cancer progression.

Emerging evidence indicates that the cancer cell-intrinsic aberration determines the immune landscape of tumors (48, 49). For example, the high mutational burden in tumors such as melanoma and NSCLC display increased T cell influx, which is associated with the beneficial outcome of checkpoint blockade treatments (50, 51). Importantly, six distinct immune subtypes have been mapped in multiple tumor types indicating that specific driver mutations dictate unique tumor microenvironments (52). Oncogenes and tumor suppressor mutations drive alterations in cancer which can metabolically modulate leukocyte function, polarization, and recruitment in the microenvironment (53, 54). Th17 cells can be generated under activation of oncogene or inhibition of tumor suppressors in both human and murine models (9–11). Oncogenic-driven NSCLC models have predominantly shown a pro-tumorigenic role of IL17A responses (9, 10), whereas IL17A regulation in a loss of tumor suppressor NSCLC model has been associated with an anti-tumorigenic function (11).

The underlying genetics of tumors can elicit the type of immune response, as shown in both the clinic and in pre-clinical models. Furthermore, the same immune milieu (e.g., TILs abundant in Th17 cells), may have opposing effects on tumor establishment and or progression. An example of this dichotomous response to IL17A has been shown in *K-ras* vs. non-*K-ras* models of NSCLC, where IL17A was shown to promote growth in *K-ras*-driven tumors (10), but IL17A were required to inhibit early oncogenesis in a non-*K-ras* driven model (11). Specifically, mice with airway specific deletion of Phosphatase and tensin homolog (*Pten*) and SMAD family member 4 (*Pts4*^*d*/*d*^) spontaneously develop NSCLC by 9 months of age (55); global deficiency of *Il17a* resulted in earlier and increased metastasis which could be rescued with adoptive transfer of IL17a-sufficient CD4^+^ T cells (11).

Th17 cell-derived cytokines can regulate stromal cells in the tumor microenvironment. IL17A promotes myeloid cell recruitment, which can suppress tumor immunity. *K-ras* mutations in alveolar epithelial cells express high levels of C-X-C motif chemokine receptor 2 (CXCR2) ligands, which recruit inflammatory and endothelial cells (56). Further, *K-ras* mutations can induce Granulocyte-macrophage colony-stimulating factor (GM-CSF) expression, which in turn recruits CD11b^+^Gr1^+^ myeloid cells and suppresses T cell immunity in tumors (57). In a *K-ras-*driven model, an oncogenic form of *K-ras* expressed in the club cells (*CCSP*^*cre*^*/K-ras*^*G*12*D*^), Th17 cells and T regulatory cells (Tregs), but not Th1 cells, were recruited to the tumor tissue (9). A mouse model of *K-ras*^*G*12*D*^ pancreatic cancer showed similar infiltration of IL17A producing cells (58). When *CCSP*^*cre*^*/K-ras*^*G*12*D*^ were induced with Chronic Obstructive Pulmonary Disease (COPD)-like inflammation, local production of IL17A recruited Gr1^+^ CD11b^+^ myeloid cells to the lung. *Il17a* deficient (*CCSP*^*cre*^*/K-ras*^*G*12*D*^*; Il17a*^−/−^) conferred decreased tumor progression, angiogenesis, pro-inflammatory cytokines, and Gr1^+^ CD11b^+^ myeloid cell abundance (9). Gr1^+^ CD11b^+^ myeloid cells, also known as myeloid derived suppressor cells (MDSCs), can inhibit the anti-tumor activity of CD8^+^ T cells. However, CD8^+^ T cells depletion did not restore the tumor growth in both IL17A sufficient and deficient conditions (9), suggesting Gr1^+^ CD11b^+^ myeloid cell can directly promote tumor growth. This concept is confirmed by depletion of Gr1^+^ CD11b^+^ cells in *CCSP*^*cre*^*/K-ras*^*G*12*D*^, resulting in reduced tumor progression (9). Similarly, IL17A depletion showed decreased CD11b^+^Gr1^+^ myeloid cells and metastasis in an oncogene-driven breast cancer model (59). IL17A-mediated induction of IL-6 and Granulocyte-colony stimulating factor (G-CSF) expression in the tumor cells has been shown to recruit tumor-associated neutrophils (Ly-6G^+^) (10). Blocking IL-6 or Ly-6G showed reduced tumor burden than anti-PD-1 treatment in high IL17A lung tumors (10). Additionally, IL17A has been shown to promote metastasis in a pre-clinical model using *K-ras-*driven NSCLC cell line, a process that was shown to be driven through IL-6 signaling (60, 61). Together, the findings in *K-ras* models indicate that IL17A is a pro-tumorigenic factor (9, 62).

**Figure 1:**
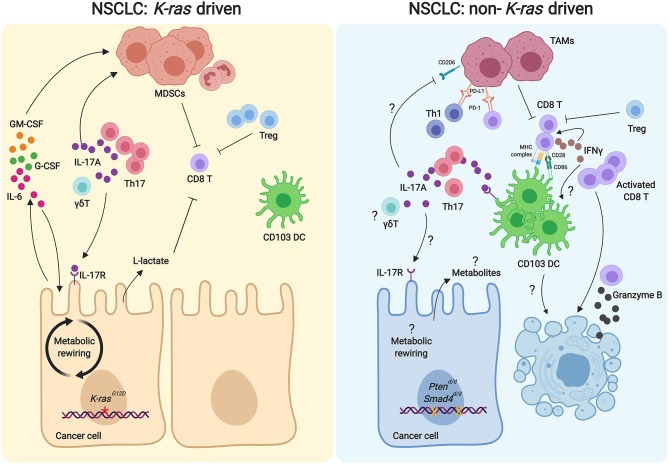
Comparison of Th17/IL17A axis in the tumor microenvironments of *K-ras*-driven and *Pts*^*d*/*d*^ NSCLC models. In the *K-ras*-driven models of NSCLC (**Left**), Th17 cells are recruited to the tumor microenvironment to promote tumor cell growth. Th17 derived IL17A recruits MDSCs to suppress the anti-tumor activity of CD8 T cells. Increased Tregs and the glycolytic metabolite lactate in the tumor microenvironment can also inhibit the CD8 T cell activation. IL17A can directly signal the tumor cells, resulting in increased IL-6 expression. IL-6 can act as an autocrine manner to foster the proliferation of tumor cells and paracrine manner to stimulate the MDSC recruitment. Additionally, GM-CSF and G-CSF produced by the tumor cells can further promote the infiltration of MDSCs. In contrast, Th17/IL17A in the tumor microenvironment of the *Pts*^*d*/*d*^ NSCLC model inhibits tumor growth (**Right**). Th17 cells are required for the recruitment of CD103^+^ DCs to activate CD8 T cells for their anti-tumor activity. IL17A induces increased expression of IL-17R and CD86 on CD103 DCs, which in turn provides signals for CD8 T cell activation. Activated CD8 T cells secrete IFNγ, which elicits CD8 T cell activation and DC tumoricidal activity to kill the tumor cells. IL17A may inhibit CD206^+^ TAMs, resulting in decreased PD-1 and PD-L1 interaction between lymphocytes and TAMs, allowing anti-tumor activity of lymphocytes. DC, dendritic cell; γδT, gamma delta T cell; GM-CSF, granulocyte macrophage colony stimulating factor; G-CSF, Granulocyte-colony stimulating factor; IFNγ, interferon gamma; IL-17R, IL-17 receptor; MDSC, myeloid derived suppressor cell; TAM, tumor associated macrophagel; Treg, regulatory T cell.

In contrast to cancer-promoting effects of IL17A described above, IL17A has been shown to repress tumor development. In a non-*K-ras* model of NSCLC model *Pts4*^*d*/*d*^ (55), Th17 cell infiltrated the lungs before- and in early-stages of NSCLC (11). Immune competent *Pts4*^*d*/*d*^ mice lacking *I117a* showed accelerated tumor progression and metastasis, indicating that IL17A plays a beneficial role in anti-tumor responses (11). Consistently, Th17 cells were also found in TILs in patients with early-stage NSCLC (11). In the *Pts4*^*d*/*d*^ model, there is an increase in Th17 cell infiltration, both before and after tumor development; however, after tumor development, there is an additional increase in the relative abundance of exhausted PD-1^+^T cells and cytotoxic T lymphocytes (CTLs) in addition to increased Th17 cells. Furthermore, a decrease in Th17 cells and an increase in PD-1^+^ T cells have been found in the mediastinal lymph nodes in both NSCLC patients and *Pts4*^*d*/*d*^ mice (11). The expression of pro-inflammatory molecules in the *Pts4*^*d*/*d*^ model has not been reported. Strikingly, in contrast to the *K-ras* model (9), the *Pts4*^*d*/*d*^ model with *Il17a*^−/−^, shows increased tumor burden and metastasis (11). Anti-tumor effects of Th17 cells found in NSCLC were also reported in the B16 melanoma model. When tumor-specific Th17 cells were adoptively transferred into the host, they induced activation of tumor-specific CD8^+^ T cell for anti-tumor effects (63). Assessment of metastases in this model revealed fewer infiltrating CTL and Th1 in metastatic sites of *Il17a*^−/−^ compared with Th17-sufficient mice, indicating that IL17A is required for efficient immune cell infiltration into metastatic sites (63). Both the B16 melanoma and *Pts4*^*d*/*d*^ NSCLC models show that anti-tumor immunity mediated by IL17A is enhanced by the recruitment of dendritic cells (DCs). IL17A can recruit CD103^+^ DCs which are critical for the anti-tumor function of CD8^+^ T cell in *Pts4*^*d*/*d*^ mice (11). *In vitro* experiments also demonstrated that IL17A directly inhibits CD206^+^ differentiation of bone marrow-derived macrophages (BMDM) and suppresses the expression of inhibitory mediators Arginase-1 and *Vegf* . IL17A increases inducible (i) CD103^+^ DC migration in a dose-dependent manner and promotes the upregulation of the co-stimulatory molecule CD86 (11). Th17 cells have also been shown to promote the recruitment of CD8α^+^ DCs in the tumor tissues in the B16 melanoma model (63). Notably, CD103^+^ DC counts were significantly fewer in the above mentioned constitutive IL17A expressed *K-ras* than *K-ras* only lung cancer model (10). Decreased CD103^+^ DC in both constitutive IL17A expressed *K-ras* and IL17A deleted *Pts4*^*d*/*d*^ models showed enhanced NSCLC progression (Figure 1). These findings further support the significance of genetic aberration in tumors that can result in a differential role for Th17 cells in NSCLC; whether and how these findings relate to immune response in other genetic mutations in cancer remains unknown. Furthermore, it remains unclear which subsets Th17 cells play pro or anti-tumor effects, and as such, this unmet need provides a new area of onco-immunity that should be explored in the future.

Cytokine milieu is not the only way to polarize Th17 cells, as metabolites are also critical for determining the cellular function and fate of immune cells (64, 65). The opposing immune responses observed in these distinct genetic models may be in part due to metabolism. Metabolic reprogramming in cancer impacts recruitment and polarization of immune cells (66, 67). The interaction between tumor cells and the surrounding stromal cells is known to be involved in cancer development and progression (66). Nutrient competition and nutrient symbiosis in the tumor microenvironment support tumor growth and attenuate anti-tumor immunity (68). Cell intrinsic genetic variables can contribute to metabolic heterogeneity in the tumor microenvironment. For example, recent findings that focused on metabolic properties of *K-ras* mutation cancer cells have shown enhanced nutrient uptake and rewiring of their metabolism (69, 70). Increased glycolysis was observed in *K-ras* NSCLC patients and a murine model (69, 71). Lactate, a product of glycolysis, is sensed by CD8^+^ T cells, disturbing their metabolism and function, resulting in decreased cell proliferation and IFN-γ expression (72). Extracellular sodium lactate can induce Th17 differentiation and IL17A expression (73). Glutamine is also a well-known nutrient for cells with high mitotic index and a potential target for cancer therapy (74). Activation of glutamine metabolism (i.e., via glutaminase) has been shown to alter chromatin and promote Th17 cell differentiation, but it constrain Th1 and CTL development (75). Future studies are needed to understand the development and the role of T cell subsets in the glutamine restricted NSCLC models. By understanding the metabolic reprogramming of cancer cells and how this metabolic shift promotes immune evasion, we can target this interaction and re-energize the anti-tumor immunity to fight cancer.

## Immunotherapy and Th17 Response in Cancer

Although an opposing role of Th17 and IL17A responses in cancer development is becoming evident, accumulating data from mouse studies suggest that Th17 cells can cause more significant tumor regression compared with Th1 cells (63, 76–78). Adoptive transfer of tumor-specific Th17 cells can mediate destruction of B16 melanoma and elicit IFN-γ production (76). Similarly, adoptive transfer of antigen-specific Th17 cells promoted CD8^+^ T cell activation *in vivo* (63). Chimeric antigen receptors (CAR)-T cells, adoptive transfer of ICOS stimulated Th17 cells, instead of CD28 stimulated Th17 cells, resulted in a robust anti-tumor activity, when compared with ICOS stimulated CD8^+^ T cells (78). These findings indicate that adoptive transfer of Th17 cells has potent anti-tumor activities. However, the exact mechanism of how adoptive transferred Th17 cells have this strong effect of activating CD8^+^ T cells and the intrinsic and extrinsic stimuli that lead to this phenotype are not clear.

Adoptive T cell therapy (ACT) has shown some efficacy in several types of cancer; however, only 25% of patients achieve durable and complete tumor regression (79). Tumor glycolysis has been associated with immune resistance to ACT where overexpression of glycolytic molecules hampered T cell-mediated killing of tumor cells (80). Tumor intrinsic genetic mutations, (e.g., *PTEN*), are the driver to promote tumor glycolysis. In clinical samples, increased glycolysis is associated with decreased T cell infiltration in melanoma and NSCLC (80), suggesting that ACT co-treated with glycolysis inhibitors may enhance the therapeutic outcome for patients. As activation of glycolysis is essential for Th17 cell development (81), investigating the efficacy of glycolysis inhibitors co-treated with Th17-based adoptive T cell therapy is critical.

Additionally, Th17 cells have stem-cell like properties which make them an ideal cell type for CAR-T cell therapy (40, 77). Although human Th17 cells express terminal differentiation markers, these cells have anti-tumor activity and long-lived *in vivo*. Furthermore, Th17 cells can self-renew and differentiate into other T helper subsets. For example, under Th1 polarizing conditions, a significant fraction of Th17 cells express IFN-γ (40, 77). *In vivo*, Foxp3^+^IL-17^+^CD4^+^ and IFN-γ^+^IL-17^+^CD4^+^T cells are present (40). Upon adoptive transfer, Th17 cells converted to Th1-like cells and produced IFN-γ, which is critical for anti-tumor effects (40, 77).

There are several concerns with targeting Th17 response in cancer, one of which is the concomitant development of Th17 cells and Tregs in cancer. The differentiation of Th17 cells is dependent on the relative expression of IL-6 and TGF-β (82). However, IL-6 inhibits Treg development (83). An appropriate balance between Th17 cells and Tregs is critical in regulating inflammation and promoting tumor immune surveillance (39, 82). Considering this delicate equilibrium between Tregs and Th17 cells in cancer, targeting Th17 cells alone may enhance the development of Tregs, and therefore suppresses antitumor activities. Targeting Th17 cell-mediated tumor-promoting inflammation and Treg-mediated immune suppression simultaneously could be an alternative way to provide effective immunotherapy in solid tumors. Checkpoint blockades that dampen active tumor immunity combined with anti-Th17 agents is another possible route to explore. However, the premise of this dual treatment is that Th17 and IL-17 responses are tumor-promoting not tumor-suppressive, which could be problematic considering the case reports mentioned above, and pre-clinical models. Therefore, while the exact mechanism(s) of immune and specific tumor responses require an individualized assessment of genes and its microenvironment, combined effects of oncogenic changes and adaptive immune therapy (e.g., Th17 cells) is an exciting consideration for near future treatment in NSCLC.

### Prospective

The summation of evidence reveals there are opposing roles for Th17 cells in different genetic drivers of cancer, though there are still some essential remaining knowledge gaps. It appears that different cancers respond distinctly to similar immune contextures. Mouse models thus far have been limited in the genetic mutations and investigation of their corresponding immune responses. Furthermore, clinical observations need to be extended to clinical studies. Future human NSCLC studies are needed to assess the immune infiltrates with different oncogenic foci. Further, how different cancers respond to, or whether tumors are reinforced or inhibited by various immune contextures, should be investigated using new sequencing techniques, and through immunogenomics of tumor samples (84–86). Genetic sequencing, mass spectrometry, and other high throughput methods can provide genetic mutation of tumor cells and the corresponding immune setting in its microenvironment (85). Therapeutically, immunogenomics is an approach to identify neo-antigens for vaccination and T cell therapy (85, 86). Determining how genetic alterations specifically affect the immune system will open up more opportunities for immunotherapy and personalized medicine. For an exploration of immunotherapy, it may be beneficial to further investigate the potential of Th17 cells in CAR-T cell therapy. Given the findings in murine models, knowing the underlying genetics of a patient's tumor may inform whether enhancing or dampening a Th17 response would improve outcomes for patients in personalized medicine.

## Author Contributions

DA and C-YC: equal contribution to conception of the work, drafting the work, final approval of version to be published, and agreement to be accountable for all aspects of the work. FK, DC, and DL: revised and edited the concepts and writing of the manuscript.

### Conflict of Interest

The authors declare that the research was conducted in the absence of any commercial or financial relationships that could be construed as a potential conflict of interest.
